# 

**Published:** 1903-04-15

**Authors:** 


### ANNOUNCEMENTS.

THE Indiana State Dental Society holds its meeting this year at Indianapolis, June 30th and July 1st and 2d. A cordial invitation is extended to all members of the profession of adjoining States.

THE Michigan Board of State Dental Examiners meets May 12th, at Grand Rapids. Applicants for registration or examination should correspond with Dr. Charles J. Gray, Secretary, Petoskey, Mich.

THE West Virginia State Board of Examiners will hold its regular spring meeting June 3rd, 4th and 5th, 1903, at Charleston, W. Va. Applicants should correspond with Dr. W. A. Williams, Secretary, Huntington, W. Va.

THE March *Items* publishes a three-page size insert-picture of a flash-light photograph taken of attendants at the great Chicago meeting of the Odontographic Society in the operating room of the Northwestern Dental School. It is a remarkable picture for one so large. Many of the faces are good likenesses.

---

Dr. Henry W. C. Bodecker, the oldest son of Dr. C. F. W. Bodecker, who now resides in Berlin, Germany, was married March 17th, in Berlin, to Miss Anna F. M. Juriawz. Henry is a graduate of the Dental and Literary Departments of the University of Michigan, and is practicing dentistry in Berlin.

---

The thirty-ninth annual meeting of the Missouri State Dental Association will be held may 19th, 20th and 21st, 1903, at Kansas City. All ethical members of the profession are invited to attend and participate. Drs. J. B. Willmott, of Toronto, Canada, E. K. Wedelstaedt, of Minneapolis, and A. C. Searle, of Owatonna, Minn., are on the program.

---

The meeting of the Institute of Dental Pedagogics has been set for Monday, Tuesday and Wednesday of December at Buffalo, this year, so that the American Odontological Society may meet at the same place the remaining three days of the same week. This will be a pleasant and agreeable combination, which, we doubt not, all interested will appreciate.

---

The Michigan Dental Association will hold its meeting July 6th, 7th and 8th, at the beautiful summer resort of Northern Michigan, Petoskey. The program will be a good one and is all promised and largely prepared. It is expected that a good time will be had in connection with the regular exercises of the meeting. Every one should plan to take in this meeting and get a supply of good pure air.

---

Mr. Samuel T. Jones, for nearly twenty-five years the treasurer of the S. S. White Dental Manufacturing Co., died January 31st, in the seventy-third year of his age. He was a valued factor in the affairs of this great and useful corporation. He entered the employ of Dr. S. S. White nearly forty-four years ago. While we knew little of him he has doubtless known us well, and must have cared for the profession to have spent so long a time in managing the important financial branch of our greatest supply house. Perhaps he contributed largely to its great success.

---

The *Medical Critic* announces that each subscriber to that journal will receive a free copy of the Medical Index, which will contain names, place and date of publication, price, circulation and names of editor and publishers of over 600 of the principal medical publications in this country and abroad, and the titles and authors of each article published during the year 1902, arranged according to subjects and alphabetically. When it is noted that the list is complete up to January 1903, it should prove especially valuable in bridging over the period which has elasped since the Index Medicus was discontinued.

Considering the expenditure of time and money in the preparation of this volume, and the liberality of the publishers in presenting it free to the profession, the enterprise marks a new era in medical journalism and merits appreciation and success.

